# Perioperative Exercise Intention and Influencing Factors: A Multi-Centered Cross-Sectional Study

**DOI:** 10.3389/fpubh.2021.653055

**Published:** 2021-05-20

**Authors:** Feng Lv, Yuxi Zhang, Su Min, Ping Li, Lihua Peng, Li Ren, Jian Yu, Bin Wang, Yiwei Shen, Shanshan Tong, Juying Jin, Xi Luo, Jing Chen, Yingrui Chen, Yuanyuan Li, Jin Chen, Xing Zeng, Fuquan Luo, Qiuju Xiong, Lei Zou, Yuanyuan Guo, Jun Cao, Qibin Chen, Bin Wu, Gang Chen, Xiaoli Liu, Boli Xie

**Affiliations:** ^1^Department of Anesthesiology, The First Affiliated Hospital of Chongqing Medical University, Chongqing, China; ^2^Department of Anesthesiology, Jiangjin Centre Hospital, Chongqing, China; ^3^Department of Anesthesiology, Yongchuan Hospital of Chongqing Medical University, Chongqing, China; ^4^Department of Anesthesiology, The People's Hospital of Liangping District of Chongqing, Chongqing, China; ^5^Department of Anesthesiology, Chongqing University Three Gorges Hospital, Chongqing, China; ^6^Department of Anesthesiology, The People's Hospital of Yubei District of Chongqing, Chongqing, China; ^7^Department of Anesthesiology, Chongqing Emergency Medical Center, Chongqing, China; ^8^Department of Anesthesiology, Youyang Hospital, The First Affiliated Hospital of Chongqing Medical University, Chongqing, China

**Keywords:** physical activity, perioperative exercise, intention, China, inpatient

## Abstract

**Objectives:** This study aimed to evaluate the level and factors affecting the perioperative exercise intention in China.

**Design:** This study was a cross-sectional survey in Southwest China.

**Methods:** Four hundred and ninety nine participants were randomly sampled in eight medical centers from November 23, 2020 to November 27, 2020. The survey included sociodemographic information and a 24-item modified questionnaire, which aimed to evaluate the attitude toward daily exercise, perception of perioperative exercise, social support and the perioperative exercise intention. A multivariable linear regression model was used to evaluate the effect of different items on the patients' intention for perioperative exercise.

**Results:** A total of 523 responses (95.09%) were collected and 499 (95.41%) were analyzed. The level of exercise intention of the patients during the perioperative period was: 14.83% planned to exercise every day in the hospital, 21.04% planned to exercise every other day, and 35.87% planned to exercise every week. Intensity of daily exercise (*P* = 0.016), positive attitude of daily exercise (*P* < 0.001), positive attitude of perioperative exercise (*P* < 0.001) and social support (*P* < 0.001) were positively associated with the intention for perioperative exercise. Female (*P* = 0.012), non-tertiary center (*P* = 0.011), and preoperative anxiety (*P* = 0.023) was negatively associated with it.

**Conclusions:** The intention for perioperative exercise was low in Southwest China. The authors aimed to relieve preoperative anxiety, promote the education of perioperative exercise, design perioperative exercise programs, and provide more social support from medical staff and family for inpatients undergoing elective surgery.

## Introduction

Physical activity (PA) is a major contributor to prevent non-communicable diseases. Strong evidence supports that PA can generate positive clinical outcomes such as reducing blood pressure, glycosylated hemoglobin, cholesterol and important positive effects on mental health. It not only provides survival benefits both in primary and secondary cardiovascular disease prevention, but also increases lung function with higher FEV_1_ and FVC (1). The World Health Organization (WHO) and American College of sports medicine PA guidelines recommend that all adults should undertake 150–300 min of moderateintensity, or 75–150 min of vigorous-intensity physical activity, or some equivalent combination of moderateintensity and vigorous-intensity aerobic physical activity, per week (2, 3). In the UK, the National Institute of Health and Care Excellence (NICE) encourages people to be physically active, and there should be referral schemes for sedentary or inactive adults (4). WHO has identified physical inactivity as the fourth leading risk factor for overall morbidity and mortality, and has adopted a new voluntary global target to reduce global levels of physical inactivity in adults and adolescents by 15% by 2030. Despite this, few adults regularly participate in PA to promote health; 31.1% of adults were physically inactive worldwide (5). It is closely related to age, gender, education, income and area (5, 6).

Temporary functional decline as a result of decreased PA is a common side-effect during the perioperative period. Surgical stress is also a physiological contributor to functional decline. A poorer preoperative physical condition could hamper post-operative recovery, increase hospitalization times and operative mortality. Our previous study and those of other researchers both show that preoperative exercise-based training can improve preoperative lung function, reduce post-operative hospital stay and post-operative complications (7–9). Early physical exercise after surgery significantly improves functional and aerobic capacity following cardiac surgery (10). Therefore, in the case of elective surgery, patients should begin physical exercise before surgery and continue to exercise early and late after surgery until the maximum recovery of physical function and social participation is achieved (11). Some recommendations on perioperative exercise training have been offered in guidelines (12, 13). In patients awaiting major non-cardiac surgery, it is recommended 30–60 min of moderate exercise, or 20–60 of vigorous exercise, or a combination of moderate and vigorous exercise per day (13).

Unlike leisure-physical exercise, perioperative exercise is affected by surgical disease, pain, mental status at the same time. However, there is a complete lack of primary data describing on the perioperative exercise intention (PEI) in surgical patients and influencing factors in China. In this paper, we report on the current profile, opinion, and factors affecting perioperative exercise in Southwest China.

## Methods

### Participants

This study was approved by the institutional Ethics Board of the First Affiliated Hospital of Chongqing Medical University (the organizing center) on November 19, 2020 (approval number: 2020-666). The study was registered before patient enrolment at http://www.chictr.org.cn (registration number: ChiCTR2000040078). A stratified random sampling survey was carried out in 8 medical centers (2 university-affiliated hospitals, 3 hospitals at provincial level, 3 secondary hospitals for adult in Southwest China) depending on geological distribution.

The study protocol was conducted according to the principles of survey research (14). Informed consent was obtained from all participants. Then inpatients undergoing elective surgery were surveyed with face-to-face on the first day of hospitalization, from November 23, 2020 to November 27, 2020. All 21 investigators were trained with the questionnaire and communication skills.

Inpatients undergoing elective surgery ranged from 18 to 90 years old. Emergency surgery, patients with cognitive dysfunction, acoustic dysfunction, visual impairment and completely self-care functional disability were not included, which was based on the patient's history; and other conditions that made it impossible for hospitalized patients to be interviewed.

### Measures and Materials

The questionnaire included eight domains with 39 items in Chinese (Appendix 1). Three experts' consensus on the suitability of the 39 items on the questionnaire set were obtained. The basic characteristics of patients included age, gender, height, weight, educational background, social and economic status, marital and children status, medical payment, self-care ability (11 items), and disease characteristics included comorbidity, preoperative pain, emotional state, surgical types (4 items). Preoperative exercise characteristics included length and intensity of daily exercise based on the Global Physical Activity Questionnaire (2 items).

The scale for measuring attitude toward daily exercise was prepared by partially modifying attitudes toward exercise scale used in older adults (15). The scale comprised 3 items (Cronbach's alpha = 0.699) with each item assessed on a 5-point Likert scale (1 = Strongly Disagree, 2 = Disagree, 3 = Uncertain, 4 = Agree, 5 = Strongly agree).

A scale for measuring perception of perioperative exercise was prepared by modifying and supplementing attitudes toward exercise scale used by Park (16). The scale comprised 10 items pertaining to perception of rehabilitation (Cronbach's alpha = 0.697), negative attitude of preoperative exercise (Cronbach's alpha = 0.699), and positive attitude of preoperative exercise (Cronbach's alpha = 0.740).

The scale to measure social support was prepared by modifying the 5 items from the study by Hankonen et al. (Cronbach's alpha = 0.850 at baseline, 0.836 in the current study) (17). To assessing PEI, authors used the scale by modifying 4 items scale from the stage models of physical activity developed by Duan (18). Cronbach's alpha was 0.677 as reported in Liu's study, and was 0.916 in this study (19).

Reliability analysis yielded a Cronbach coefficient alpha of 0.873 for the total sample.

### Sample Size Calculation

A previous study reported that percentage of persons with low physical activity was ~50% in China (20). The requirement of minimum sample size in this study was 402 participants to reach the statistical significance at two-sided 95% confidence interval with a width equal to 0.100 when the sample proportion is 0.500. In addition, the total sample size was 503 by assuming a 20% attrition during follow-up. Finally, the scheduled sample was decided to be 550. The sample size calculation was performed PASS 15.0 analysis program.

### Statistical Strategies

The responses to the questions were summarized and analyzed via SPSS 21.0. Baseline characteristics were described by descriptive statistics. The continuous variables were compared by *T*-test (parametric)/Mann–Whitney U test (non-parametric) between two independent samples, and compared by One-Way ANOVA (parametric)/Kruskall-Wallis (non-parametric) tests between more than two independent samples. Pearson's (parametric)/Spearman's (non-parametric) tests were used to assess the correlation between continuous variables. The internal reliability of the training satisfaction questionnaire was assessed by Cronbach's alpha. A multivariable linear regression model was used to evaluate the effect of different items on PEI. The graphs were created by Graphpad prism 8. A value of *P <* 0.05 was considered statistically significant.

## Result

**Figure 1:**
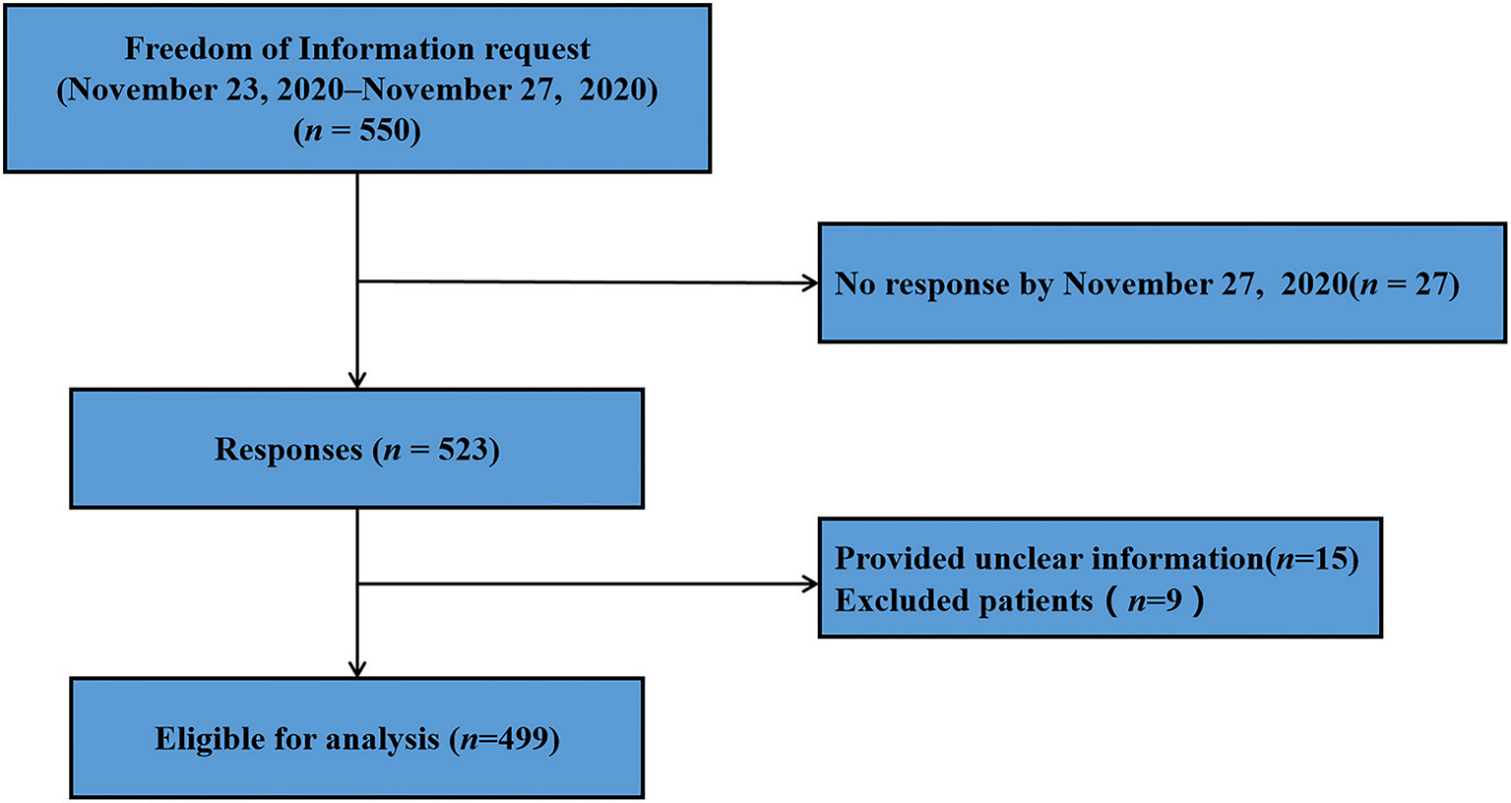
Flowchart of the study population selection.

A total of 550 questionnaires were send to different medical centers, 523 patients had been completed by November 27, 2020, with a response rate of 95.09%. Of these, we excluded 15 patients and 9 patients did not meet the inclusion criteria, therefore, a total of 499 patients were included in this analysis (Figure 1). The face-to-face interview for patients took 278.00 (interquartile range, IQR, 288) s to complete the questionnaire in this survey.

The baseline characteristics of patients are described in Table 1. The range of age were 18–90 years, the median age was 53 years (IQR, 20). 46.89% of the patients were male and 53.11% were female. Overall, 4.61% of the patients had a body mass index (BMI) < 18.5 kg/m^2^, and 42.28% had a BMI > 24.0 kg/m^2^. The education level of the patients was mainly primary and middle school (64.53%), undergraduate and graduate students accounted for only 15.83%, and working patients accounted for 72.34%. 10.82% of the patients had a monthly income of more than 10,000 yuan. A majority of the patients complained of mild pain before surgery (43.29%), and 15.43% reported moderate or severe pain. Most patients (69.74%) could meet the WHO PA recommendation (moderate intensity PA of 150 min, or 75 min vigorous-intensity PA per week). 83.57% of the patients took exercise at a moderate-vigorous intensity in daily life. The majority of the patients felt anxiety before surgery (79.16%).

**Table 1:** Baseline characteristics of survey respondents and factors affecting overall perioperative exercise intention (*N* = 499).

**Characteristics**	***n***	**(%)**	**Perioperative exercise intention Median (IQR)**	**Statistical methods**	***P-*value**
**Gender**					
Male	234	(46.89%)	11.00 (4)	Mann–Whitney U test	0.068
Female	265	(53.11%)	10.00 (4)		
**Age (yr)**					
18–35	77	(15.43%)	11.00 (5)	Kruskall-Wallis test	0.331
36–49	115	(23.05%)	12.00 (4)		
50–59	141	(28.25%)	10.00 (5)		
≥60	166	(33.27%)	11.00 (4)		
**BMI (kg/m**^**2**^**)**					
<18.5	23	(4.61%)	12.00 (3)	Kruskall-Wallis test	0.066
18.5–23.9	255	(51.10%)	10.00 (3)		
≥24.0	221	(42.28%)	11.00 (5)		
**Educational background**					
Primary school	165	(33.07%)	10.00 (3)	Kruskall-Wallis test	0.047
Middle school	157	(31.46%)	10.00 (4)		
High school	101	(20.24%)	12.00 (5)		
Bachelor or post-graduate	76	(15.83%)	11.00 (4)		
**Social status**					
Employed	361	(72.34%)	10.00 (3)	Mann–Whitney U test	0.001
Retried or unemployed	138	(27.66%)	12.00 (6)		
**Monthly incomes(RMB, Yuan)**					
≤1,000	100	(20.04%)	9.00 (4)	Kruskall-Wallis test	0.001
1,001–3,449	182	(36.47%)	11.00 (4)		
3,500–9,999	163	(32.67%)	11.00 (4)		
≥10,000	54	(10.82%)	12.50 (7)		
**Marital status**					
Married	453	(90.78%)	11.00 (4)	Mann–Whitney U test	0.122
Single or divorced	46	(9.22%)	10.00 (4)		
**Children status**					
Have children	461	(92.38%)	11.00 (4)	Mann–Whitney U test	0.852
No children	38	(7.62%)	10.50 (4)		
**Medical payment**					
Medical insurance	462	(92.59%)	11.00 (4)	Mann–Whitney U test	0.970
Self-paying	37	(7.41%)	11.00 (5)		
**Self-care ability**					
Partly capable	116	(23.25%)	10.00 (3)	Mann–Whitney U test	0.041
Completely capable	383	(76.75%)	11.00 (4)		
**Comorbidity**					
Yes	243	(48.70%)	11.00 (3)	Mann–Whitney U test	0.933
No	256	(51.30%)	11.00 (4)		
**Preoperative pain**					
No pain	206	(41.28%)	11.00 (4)	Kruskall-Wallis test	0.813
Mild	216	(43.29%)	11.00 (4)		
Moderate	63	(12.62%)	11.00 (5)		
Severe	14	(2.81%)	12.00 (4)		
**Surgical types**					
Head or neck surgery	62	(12.42%)	12.00 (7)	Kruskall-Wallis test	0.061
Thoracic surgery	36	(7.21%)	12.00 (5)		
Abdominal surgery	273	(54.71%)	11.00 (4)		
Limb surgery	99	(19.84%)	10.00 (5)		
Spinal or brain surgery	29	(5.81%)	9.00 (4)		
**75 min of vigorous or 150 min of moderate intensity per week**					
Yes	348	(69.74%)	11.00 (4)	Mann–Whitney U test	0.742
No	151	(30.26%)	11.00 (3)		
**Intensity of daily exercise**					
Light	82	(16.43%)	10.50 (4)	Kruskall-Wallis test	0.224
Moderate	266	(53.31%)	11.00 (4)		
Vigorous	151	(30.26%)	11.00 (6)		
**Preoperative anxiety**					
Yes	395	(79.16%)	10.00 (4)	Mann–Whitney U test	0.000
No	104	(20.84%)	12.00 (5)		
**Hospital grade**					
Tertiary hospital	370	(74.15%)	12.00 (5)	Mann–Whitney U test	0.000
Non-tertiary center	129	(25.85%)	9.00 (2)		

*BMI, body mass index; IQR, interquartile range*.

**Figure 2:**
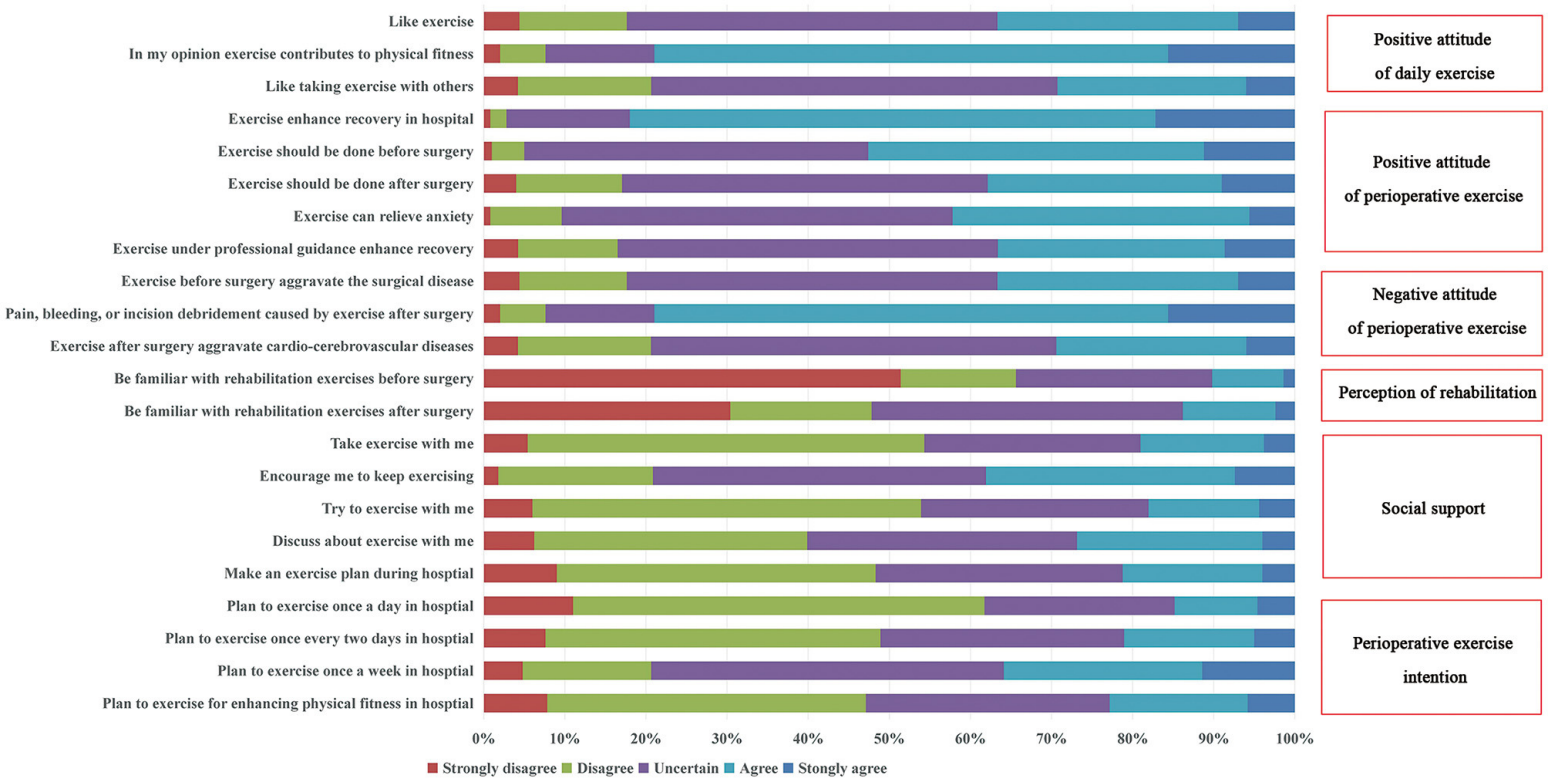
Respondents' perioperative exercise intention (scores from 4 to 20), positive attitude of daily exercise (scores from 3 to 15), positive attitude of perioperative exercise (scores from 5 to 25), negative attitude of perioperative exercis (scores from 3 to 15), perception of rehabilitation (scores from 2 to 10), and social support (scores from 5 to 25).

The scores in PEI items were from 5 to 20, the median was 11.00 (IQR, 4). Only 74 (14.83%) of the patients planned to take exercise every day in hospital, 105(21.04%) once every 2 days and 179 (35.87%) per week (Figure 2). A comparison of PEI scores between these various factors found that they differed significantly in educational background (*P* = 0.047), social status (*P* = 0.001), monthly incomes (*P* = 0.001), self-care ability (*P* = 0.041), preoperative anxiety (*P* < 0.001), and hospital grade (*P* < 0.001) (Table 1).

In terms of their attitude toward daily sports, 394 people (78.96%) believed that daily sports were beneficial to their health, only 183 people (36.67%) liked sports, and 146 people (29.26%) were willing to do sports with others. Most of the patients believed that exercise could enhance the recovery in hospital (82.16%), and exercise should be done before (52.70%) or after (37.88%) surgery. 42.28% of the patients thought exercise could relieve anxiety, and 36.47% of the patients thought exercise under professional guidance could promote recovery. More than three quarters of the patients were concerned that exercise would increase the risk of post-operative pain, bleeding, or wound debridement (78.96%), as well as exacerbate preoperative surgical disease (36.67%) and post-operative cardiovascular and cerebrovascular disease (29.46%). The patients were unfamiliar with rehabilitation exercises before (10.22%) and after (13.83%) surgery. In this survey, social support from family or medical staff movements was low (Figure 2).

**Figure 3:**
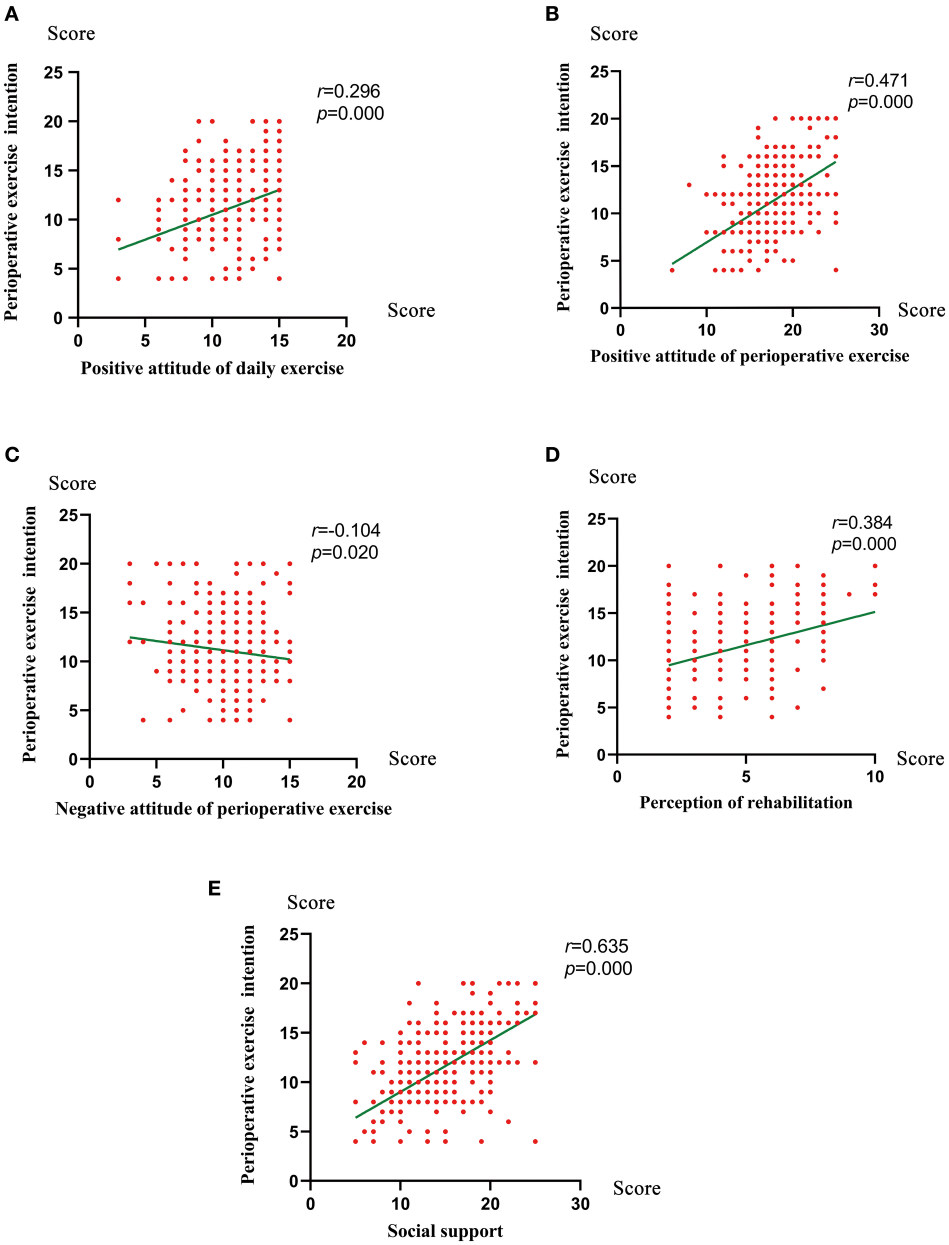
The correlation between the perioperative exercise intention and positive attitude of daily exercise **(A)**, positive attitude of perioperative exercise **(B)**, negative attitude of perioperative exercise **(C)**, perception of rehabilitation **(D)**, and social support **(E)** used by Spearman's tests.

Figure 3 showed a higher PEI was associated with positive attitude of daily exercise (*r* = 0.296, *P* < 0.001),positive attitude of perioperative exercise (*r* = 0.471, *P* < 0.001), perception of rehabilitation (*r* = 0.284, *P* < 0.001), social support (*r* = 0.635, *P* < 0.001) and was negatively correlated with negative attitude of perioperative exercise (*r* = −0.104, *P* = 0.020). The variables which remained significantly associated with PEI on multivariable regression analysis included gender (*P* = 0.012), intensity of daily exercise (*P* = 0.016), hospital grade (*P* = 0.011), positive attitude of daily exercise (*P* < 0.001), preoperative anxiety (*P* = 0.023), positive attitude of perioperative exercise (*P* < 0.001), and social support (*P* < 0.001) (Table 2).

**Table 2:** Multivariate liner regression analysis for perioperative exercise intention (*N* = 499).

**Model**	**β**	***SE***	***β'***	***t***	***P-*value**	**95% confidence interval for** ***β***	
						**Lower bound**	**Upper bound**
Female	−0.598	0.237	−0.088	−2.521	0.012	−1.064	−0.132
Intensity of daily exercise	0.446	0.186	0.088	2.425	0.016	0.085	0.808
Non-tertiary center	−0.808	0.315	−0.104	−2.568	0.011	−1.426	−0.190
Positive attitude of daily exercise	0.232	0.061	0.142	3.782	0.000	0.111	0.352
Preoperative anxiety	−0.659	0.289	−0.079	−2.285	0.023	−1.226	−0.092
Positive attitude of preoperative exercise	0.200	0.047	0.170	4.276	0.000	0.108	0.292
Social support	0.388	0.033	0.470	11.653	0.000	0.323	0.454

## Discussion

This is the first survey on PEI of surgical inpatients and influencing factors in China. Our results demonstrated that there is a low level perioperative exercise intention Chinese people, which is affected by multiple factors including biological and social environment. An overall Cronbach's alpha = 0.86 of inpatient responses shows that the items on PEI have a high internal consistency.

Regular physical activity helps to improve physical and mental functions and helps individuals to maintain a healthy weight as well as reverse some effects of chronic disease. Globally, 81.0% of students aged 11–17 years were insufficiently physically active, (21) while 31.1% of adults were physically inactive (5) and 35% of older adults did not reach the global physical activity recommendations (22). Perioperative exercise could improve physical fitness and health status and reduce the risk of peri-operative morbidity and mortality. The percentage of planning to exercise every day in hospital was just 14.83% in this study.

According to the findings of this study, women are less likely to exercise in hospital than men. Studies conducted in many countries have reported less PA among women than men (23, 24). PA levels gradually decline with age, and the decline is greater in women (25). The standard of living, work status, smoking status and social support contribute to the gender difference (17, 26). So we should be more concerned and give much support for women exercise.

Among older adults, an age related decrease in the intensity of physical activity was associated with a higher risk of depressive symptoms (27). Among adults and older adults, those who met WHO guidelines for moderate and vigorous exercise, but also used both, reported significantly higher levels of happiness (28). Our findings documented that moderate-intensity activity was the most beneficial activity level for improving PEI.

There is a significant association between socioeconomic position and physical activity (6, 29). Compared with rural areas and commuters, urban, educated and affluent people are more active in physical activity in their leisure time, but less active at work (6). In a study of twins over a 35-year follow-up study, the higher education was associated with lower odds of leisure-time physical inactivity (30). We did not find that the direct relationship between the socioeconomic position and PEI, but the patients in tertiary hospital had higher intention to PEI in this study. The reason may be that patients with high income and education background are easier to have their surgeries in tertiary hospitals with more social and medical resources in China.

Attitudes and beliefs about PA and health have been reported to correlate with PA levels (31). According to our findings, the patients with positive attitude of daily and perioperative exercise were more likely to take exercise during hospital. Inpatients appear to have a negative attitude toward physical activity, mainly due to the lack of counseling provided by medical staff (32). Social support from family and medical staff significantly impact the PEI in this study. Among patients with coronary artery disease, social support from medical staff plays a key role in promoting physical activity and concern by families and physicians about the relative risk of kidney disease was inversely associated with patient inactivity (32). Therefore, it could be recommended that family and medical staff should spend more time and put more effort in informing and counseling the patients toward increased perioperative exercise.

A perspective from the world health survey demonstrates that anxiety is associated with less physical activity, 33.1% of 24,850 people with anxiety symptoms were at low PA (33). Anxiety is one of the most common psychological reactions observed in patients waiting for various types of surgery, even occurring in up to 80% of patients (34). Our data showed that the prevalence of preoperative anxiety was 79.16%, which reduced the PEI. The risk factors of preoperative anxiety included female, having a higher American Society of Anesthesiologists (ASA) grading, psychiatric illness, high baseline anxiety levels, previous adverse clinical experiences, and undergoing specific types of operation. Preoperative anxiety could be reduced by reading a procedural information leaflet and receiving preoperative behavioral training (35).

To the best of our knowledge, this is the first cross-sectional study to examine perioperative motor intention and its influencing factors. Despite a high response rate in this survey, some limitations and avenues for future research should be noted. Firstly, investigated the perioperative exercise intention by questionnaire responses, and further observation and randomized controlled studies will be useful in future. Secondly, due to the particularity of perioperative exercise, although many scales have been used in the investigation of daily exercise, the development and validation of perioperative exercise scale still needs more research support. Thirdly, the influencing factors of perioperative movement need to be further observed and analyzed.

## Conclusion

This survey provides a contemporary assessment of the current intention for perioperative exercise of patients undergoing elective surgery in Southwest China. The intention for perioperative exercise should consider the importance of gender, intensity of daily exercise, hospital grade, positive attitude of daily exercise, preoperative anxiety, positive attitude of perioperative exercise and social support. Therefore, this study is a call to action to relieve preoperative anxiety, promote the education of perioperative exercise, design perioperative exercise programs and provide more social support from medical staff and family. In the future research and clinical practice, in-depth interviews, observational studies and clinical trials should be conducted to further explore the patients' intention for perioperative exercise and its influencing factors.

## Data Availability Statement

The raw data supporting the conclusions of this article will be made available by the authors, without undue reservation.

## Ethics Statement

The studies involving human participants were reviewed and approved by the institutional Ethics Board of the First Affiliated Hospital of Chongqing Medical University (approval number: 2020-666). The patients/participants provided their written informed consent to participate in this study.

## Author Contributions

SM, FLv, YZ, and LR: study design. YS, ST, JJ, BWa, XLu, JingC, YC, YL, JinC, XZ, FLu, QX, LZ, LP, YG, JCa, QC, BWu, GC, XLi, and BX: data collection. FLv, YZ, LR, JY, and YS: statistical analysis. FLv, YZ, LR, and JY: drafting of the manuscript. SM, FLv, YZ, LR, PL, LP, and BWa: manuscript preparation. All authors edited, read, and approved the final version of the manuscript.

## Conflict of Interest

The authors declare that the research was conducted in the absence of any commercial or financial relationships that could be construed as a potential conflict of interest.
