# Supplementary material for: Perioperative Exercise Intention and Influencing Factors: A Multi-Centered Cross-Sectional Study
Source: Front Public Health. 2021 May 20;9:653055. doi: 10.3389/fpubh.2021.653055 (PMC8172588; doi:10.3389/fpubh.2021.653055)
Supplement: Supplementary file 1 [file Data_Sheet_1.docx]

**Appendix 1**

| 1. Your gender | Male Female | | | | |
| --- | --- | --- | --- | --- | --- |
| 1. Your age | Years | | | | |
| 1. Your height | cm | | | | |
| 1. Your weight | kg | | | | |
| 1. Your education background | Primary school Middle school High school Bachelor or postgraduate | | | | |
| 1. Social status | Employed Retried or unemployed | | | | |
| 1. Monthly incomes(RMB,Yuan) | ≤1000 1001-3449 3500-9999 ≥10000 | | | | |
| 1. Marital status | Married Single or divorced | | | | |
| 1. Children status | Have children No children | | | | |
| 1. Medical payment | Medical insurance Self-paying | | | | |
| 1. Self-care ability | Partly capable Completely capable | | | | |
| 1. Comorbidity | Yes No | | | | |
| 1. Preoperative pain | 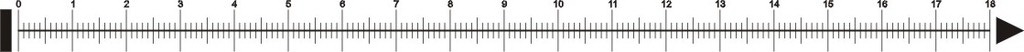  No pain 0 Mild 1-3; Moderate 4-6; Severe 7-10 | | | | |
| 1. Surgical types | Head or neck surgery Thoracic surgery Abdominal surgery  Limb surgery Spinal or brain surgery | | | | |
| 1. Intensity of daily exercise | Light(walking for at least 10 minutes continuously)  Moderate ( brisk walking,carrying light loads,jogging, climb the stair, skating, volleyball, swimming, cycling for at least 10 minutes continuously)  Vigorous(carrying or lifting heavy loads, digging or construction work,running, football, basketball,boxing for at least 10 minutes continuously) | | | | |
| 1. 75min of Vigorous or 150min of moderate intensity per week | Yes No | | | | |
| 1. Do you think you are anxiety before the operation? | Yes No | | | | |
|  | **Attitude of daily exercise** | | | | |
|  | Strongly Disagree | Disagree | Uncertain | Agree | Strongly agree |
| 1. Do you like to exercise? | 1 | 2 | 3 | 4 | 5 |
| 1. Do you think exercise contributes to physical fitness? | 1 | 2 | 3 | 4 | 5 |
| 1. Do you like taking exercise with others? | 1 | 2 | 3 | 4 | 5 |
|  | **Negative attitude of preoperative exercise** | | | | |
|  | Strongly Disagree | Disagree | Uncertain | Agree | Strongly agree |
| 1. Are you worried about that exercise before surgery will aggravate the surgical disease? | 1 | 2 | 3 | 4 | 5 |
| 1. Do you worry about pain, bleeding, or incision debridement caused by exercise after surgery? | 1 | 2 | 3 | 4 | 5 |
| 1. Do you worry that exercise after surgery may aggravate cardio-cerebrovascular diseases? | 1 | 2 | 3 | 4 | 5 |
|  | **Positive attitude of preoperative exercise** | | | | |
|  | Strongly Disagree | Disagree | Uncertain | Agree | Strongly agree |
| 1. Do you think exercise will enhance recovery in the hospital? | 1 | 2 | 3 | 4 | 5 |
| 1. Do you think exercise should be done before the operation instead of staying in bed? | 1 | 2 | 3 | 4 | 5 |
| 1. Do you think exercise should be done after the operation instead of staying in bed? | 1 | 2 | 3 | 4 | 5 |
| 1. Do you think exercise can help relieve anxiety? | 1 | 2 | 3 | 4 | 5 |
| 1. Do you think exercise under professional guidance before and after surgery can enhance the recovery of surgery? | 1 | 2 | 3 | 4 | 5 |
|  | **Perception of rehabilitation** | | | | |
|  | Strongly Disagree | Disagree | Uncertain | Agree | Strongly agree |
| 1. Have you ever heard of rehabilitation exercises before surgery? | 1 | 2 | 3 | 4 | 5 |
| 1. Have you ever heard of rehabilitation exercises after surgery? | 1 | 2 | 3 | 4 | 5 |
|  | **Social support** | | | | |
| After admission, your family or medical staff | Strongly Disagree | Disagree | Uncertain | Agree | Strongly agree |
| 1. Take exercise with me | 1 | 2 | 3 | 4 | 5 |
| 1. Encourage me to keep exercising | 1 | 2 | 3 | 4 | 5 |
| 1. Try to exercise with me | 1 | 2 | 3 | 4 | 5 |
| 1. Discuss about exercise with me | 1 | 2 | 3 | 4 | 5 |
| 1. Make an exercise plan during the hospital stay | 1 | 2 | 3 | 4 | 5 |
|  | **Perioperative exercise intention** | | | | |
| 30-60 min·day^-1^ of moderate exercise, or 20-60 min·day^-1^of vigorous exercise, or a combination of moderate and vigorous exercise per day^[1]^ | Strongly Disagree | Disagree | uncertain | Agree | Strongly agree |
| 1. I plan to exercise every day in hospital | 1 | 2 | 3 | 4 | 5 |
| 1. I plan to exercise once two days in hospital | 1 | 2 | 3 | 4 | 5 |
| 1. I plan to exercise once a week in hospital | 1 | 2 | 3 | 4 | 5 |
| 1. I plan to exercise for enhancing physical fitness in hospital | 1 | 2 | 3 | 4 | 5 |

**References**

[1] Tew GA, Ayyash R, Durrand J, Danjoux GR. Clinical guideline and recommendations on pre-operative exercise training in patients awaiting major non-cardiac surgery. Anaesthesia. 2018. 73(6): 750-768.
